# How does the EQ-5D-Y Proxy version 1 perform in 3, 4 and 5-year-old children?

**DOI:** 10.1186/s12955-020-01410-3

**Published:** 2020-05-24

**Authors:** Janine Verstraete, Andrew Lloyd, Des Scott, Jennifer Jelsma

**Affiliations:** 1 Department of Paediatrics and Child Health, Division of Medicine, Klipfontein Road, Rondebosch, Cape Town, 7700 South Africa; 2 Acaster Lloyd Consulting Ltd., London, UK; 3 Faculty of Health and Rehabilitation Sciences, Division of Physiotherapy, Anzio Road, Observatory, Cape Town, 7925 South Africa

**Keywords:** Health Related Quality of Life, HRQoL, Proxy, EQ-5D-Y, child, pre-school

## Abstract

**Background:**

The EQ-5D-Y Proxy is currently recommended for Health Related Quality of Life (HRQoL) measurement in children aged 4–8 years of age. However, it has only been validated in children over six years of age. The aim of this study was to investigate the performance of the EQ-5D-Y proxy version 1 in children between the ages of 3–6 years.

**Methods:**

A sample of 328 children between 3 and 6 years of age were recruited which included children who were either acutely-ill (AI), chronically-ill (CI) or from the general school going population (GP). The EQ-5D-Y Proxy Version 1 and the PedsQL questionnaires were administered at baseline. The EQ-5D-Y Proxy was administered telephonically 24 h later to children with chronic illnesses to establish test-retest reliability. The distribution of dimensions and summary scores, Cohen’s kappa, the intraclass correlation coefficient, Pearson’s correlation and Analysis of variance were used to explore the reliability, and validity of the EQ-5D-Y for each age group. A single index score was estimated using Latent scores and Adult EQ-5D-3 L values (Dolan).

**Results:**

The groups included 3-year olds (*n* = 105), 4-year olds (*n* = 98) and 5-years olds (*n* = 118). The dimension *Looking after Myself* had the greatest variability between age groups and had the highest rate of problems reported. *Worried, Sad or Unhappy* and *Pain or Discomfort* were not stable across time in test-retest analysis. The Visual Analogue Scale (VAS), and single index scores estimated using the latent values and Dolan tariff had good test retest (except for the latent value scores in a small number of 4-year olds). EQ-5D-Y scores for all ages had small to moderate correlations with PedsQL total score. The EQ-5D-Y discriminated well between children with a health condition and the general population for all age groups. Caregivers reported difficulty completing the *Looking after Myself* dimension due to age-related difficulties with washing and dressing.

**Conclusion:**

The dimension of *Looking after Myself* is problematic for these young children but most notably so in the 3 year old group. If one considers the summary scores of the EQ-5D-Y Proxy version 1 it appears to work well. Known group validity was demonstrated. Concurrent validity was demonstrated on a composite level but not for individual dimensions of *Usual Activities or Worried, Sad or Unhappy.*. The observable dimensions demonstrated stability over time, with the inferred dimensions (*Pain or Discomfort and Worried, Sad or Unhappy*) less so, which is to be expected. Further work is needed in exploring either the adaptation of the dimensions in the younger age groups.

## Introduction

A key focus of the World Health Organisation (WHO) and its member states is the improvement of child health globally [1]. New measures of child health and health related quality of life (HRQoL) have been developed over the last two decades [2, 3]. HRQoL measures aim to capture the subjective multi-dimensional constructs of HRQoL namely physical, social and psychological functioning which are relevant to health [4, 5].

By definition, an individual’s HRQoL is subjective and should be elicited by self-report whenever possible, even from children [6]. This is not always possible as there are those who are either too young or cognitively unaware to self-report and so it is necessary to rely on proxy report [6–8]. For younger children below the age of 7–8 years it is usually necessary to rely on proxy report [9].

The EQ-5D-Y was developed and validated for children and adolescents aged 8–18 years by an international team from the EuroQol group [10]. The youth version of the instrument was derived from the EQ-5D-3 L, an adult HRQoL instrument which is often used to support economic evaluation. The EQ-5D-Y includes five dimensions which are similar to the original EQ-5D but adapted for children and a Visual Analogue Scale (VAS) for an overall rating of health on a scale, with 0 indicating worst health imaginable and 100 indicating best imaginable health. Although a protocol has been developed for the valuation of the EQ-5D-Y a valuation set is not yet available [11]. A latent scoring system has been previously developed using a discrete choice experiment (DCE) which will be used as a composite score in this study [12].

The EQ-5D-Y Proxy is a direct adaptation of the EQ-5D-Y for proxy completion and is currently recommended by the EuroQoL Foundation for use in children aged 4–8 years and older children if they cannot complete the forms themselves [13]. The proxy version has been validated in a Spanish study in children over 6 years of age [14]. However, much less is known about the psychometric performance of the measure in younger children. This study aims to test the psychometric properties of the EQ-5D-Y Proxy version for children in three age groups: 3 years, 4 years and 5 years. The study was designed to explore if the psychometric performance of the measure is systematically worse when used with younger children. This then could provide an empirical basis for recommending which children the measure can be used with.

## Methodology

### Participants

Children aged 3–6 years were recruited from a paediatric hospital and pre-primary schools in Cape Town, South Africa. The paediatric hospital admits acutely-ill (AI) children to the in-patient facility and manages chronically-ill (CI) children in the out-patient facility. The pre-primary schools accept children from the general population (GP), some of whom may have minor health conditions. The HRQoL data were collected from their primary caregivers (typically a parent).

Recruitment was restricted to caregivers who were literate in English (due to the unavailability of translations of some the instruments into the local languages). Children who were medically unstable or critically ill in the intensive care unit were excluded.

### Measures

#### Demographic and medical information

The survey also included background questions to record age, gender, diagnosis and relationship of caregiver to child. Caregivers were asked whether each of the EQ-5D-Y dimension questions was suitable for the age of their child and, if not, to please provide an explanation.

#### EQ-5D-Y Proxy version 1

The EQ-5D-Y Proxy version 1 includes five dimensions: *Mobility* (walking about), *Looking after Myself* (washing and dressing), *Usual Activities*, *Pain or Discomfort* and *Worried, Sad or Unhappy*. Each item has three response levels corresponding to ‘no problems’, ‘some problems’ and ‘a lot of problems’. Participants are also asked to rate the global health of the child on a Visual Analogue Scale (VAS) from worst imaginable health (0) to best imaginable health (100) [15, 16]. Proxy version 1, which asks the respondent to rate the child’s HRQoL from their own viewpoint was used in this study [17]. A beta telephone-based EQ-5D-Y Proxy version 1 was used for repeat assessments which includes a telephonic script for interviewer to ensure standardisation for completion. The EuroQoL Group defines a Beta version as one that is in the final stages of development but is not yet recognized as an official version.

#### Pediatric Quality of Life Inventory (PedsQL)

The PedsQL is a widely used HRQoL measure with proxy versions for children as young as 2 years of age [18]. The PedsQL consist of four dimensions of functioning: physical, emotional, social and school with 8,5,5 and 5 items respectively. Each item is scored on a Likert scale from 0 to 4 (never a problem to almost always a problem).. Items are reversed scored and transformed to a 0–100 scale: 0 = 100, 1 = 75, 2 = 50, 3 = 25, 4 = 0. Dimension scores are calculated by a sum of the item scores divided by the total number of items. A total score is similarly generated by summing the dimension scores over the total number of dimensions giving an overall HRQoL score. A higher PedsQL score indicates a better HRQoL. The PedsQL is a profile measure which has been utilised previously to explore the concurrent validity of the EQ-5D-Y [19–21].

### Procedure

Ethical approval for the study was granted by the Human Research Ethics Committee of the Faculty of Health Sciences, University of Cape Town (HREC/REF: 825/2017) and approval was gained from all relevant authorities. The study plan was also reviewed and approved by the EuroQol Group. Children were recruited during either routine outpatient visits or from the in-patient facility at the children’s hospital. Children from the general population were recruited from pre-schools during a pre-arranged period with the school and caregivers.

The purpose and procedure of the study were explained to the parents/caregivers by one of the researchers and informed consent was obtained from those who indicated willingness to participate. Caregivers of CI children were asked to provide a repeat telephone-based assessment after 24 h to determine the test-retest reliability. The same caregiver was asked to answer the repeat telephonic measure of the EQ-5D-Y Proxy for that day as per the telephonic script, both the caregiver and interviewer were blinded to previous responses. There are no current guidelines on the time period of test-retest reliability and Marx et al. (2003) have found no difference between 2 days and 2 weeks [22]. Due to the heterogeneity of the CI sample a time period of 24 h was selected to ensure that no health-related changes occurred with repeat measurement. GP children were not included for test-retest as we expect them to report no problems in most dimensions with little variance for test-retest reliability.

A detailed description of the study, informed consent and the research pack (EQ-5D-Y Proxy, PedsQL and background questionnaire) were sent home with each of the children attending the pre-schools. The caregivers were given 1 week in which to provide informed consent and to complete the research pack.

### Data analyses and management

The sample size was powered to detect a difference in proportions across the three age bands. The degrees of freedom were thus [2 groups (GP and those with a health condition) -1] + [5 levels − 1] =5. It was anticipated that the effect size of the age bands would be small, i.e. 0.3. A minimum total sample of 220 children, i.e. 220 GP and children with a health condition was required to ensure a power of 95% with a significance level of 0.05.

Participants were grouped according to age groups based on their birthday. The EQ-5D-Y responses were summarised in terms of frequency of responses to each dimension across the age categories. Single index score were calculated using both the adult EQ-5D-3 L United Kingdom (Dolan) tariff [23] and the EQ-5D-Y summary latent value [12]. The Dolan Tariff is valued between − 0.594 and 1.000 with a higher value indicating a better HRQoL. Similarly, the Latent scale is valued between − 9.306 and 0 with a higher value indicating a better HRQoL. Test-retest reliability was assessed using the kappa statistic for dimension scores and the Intraclass Correlation Coefficient (ICC) for summary scores. Kappa values were interpreted according to Landis and Koch’s guidelines with kappa < 0.2 poor agreement, 0.21–0.40 fair agreement, 0.41–0.60 moderate agreement, 0.61–0.80 substantial agreement, and kappa > 0.81 indicating almost perfect agreement [24].. An ICC of > 0.7 was considered reliable [25]. The concurrent validity of the dimension scores of the PedsQL and EQ-5D-Y was determined using the Partial Eta Squared. Interpretation of Partial eta-squared is: small effect (0.01), medium effect (0.06) and large effect (0.14) [26] . Pearson’s r was used to explore the concurrent validity between EQ-5D-Y dimension summary scores (Latent value, Dolan tariff) and EQ-5D-Y VAS and summary scores on the and PedsQL. For known-group analysis children who were AI and CI were combined into a group labelled health condition for comparison to those who were from the general population across the age groups. As the group of AI and CI children were heterogenous expected differences between AI and CI could not be hypothesised, it was however expected that children with a health condition would report worse HRQoL than those without. The known-group validity was assessed for the mean Latent score, Dolan tariff and the VAS groups across the age groups by computing the Analysis of Variance (ANOVA).

## Results

### Descriptive Statistics

A total of 328 children and caregivers were recruited from a tertiary paediatric hospital and schools in the same geographical area that the hospital serves. All 229 caregivers approached at the paediatric hospital agreed to participate and no one was excluded due to lack of English literacy. The three English medium schools identified 156 children who were aged between 3 and 6 years. Research packs were sent out to all 156 caregivers of which 92 returned signed consent and the research packs. Data from seven children in the GP group were excluded because more than three dimensions on the EQ-5D-Y or PedsQL were not completed. The data of 321 children has been included for analysis. The participants were categorized by age and in terms of AI, CI or GP. Most proxy respondents across age groups were mothers and other caregivers included grandparents, foster parents, adoptive parents and a sister (Table 1).

**Table 1:** Descriptive statistics of the sample

Age group		3 years (***n*** = 105)	4 years (***n*** = 98)	5 years (***n*** = 118)	Total (***n*** = 321)
		N (%*)	N (%*)	N (%*)	N (%**)
**Relationship of caregiver to child**	Mother	88 (84)	77 (79)	93 (79)	257 (80)
	Father	12 (11)	17 (17)	13 (11)	42 (13)
	Other	5 (5)	4 (4)	12 (10)	22 (7)
**Gender of Child**	Female	47 (45)	54 (55)	50 (42)	151 (47)
**Health Condition of child**					
*Acutely-ill*		41 (39)	46 (47)	41 (35)	128 (40)
*Chronically-ill*		32 (30.5)	23 (23)	46 (39)	101 (31)
*General population*		32 (30.5)	29 (30)	31 (26)	92 (29)

*% of age group, **% of total sample. The chi-square statistic is 2.024; *p*-value 0.730 for health condition of the child across age groups. The result is not significant at *p* < 0.05

The presenting conditions of AI children included general surgery, systemic infection, respiratory infection, fractures and burn wounds. The CI children were diagnosed with either cerebral palsy, cancer or a respiratory disease.

Although there were a higher percentage of children in the 4-year-old group who were AI, the distribution of health conditions across age groups was not significantly different (*p* = 0.73).

### General Instrument Performance

At baseline assessment there were 321 completions of the EQ-5D-Y included for analysis. There were two missing responses in the dimension of *Mobility*.

**Fig. 1:**
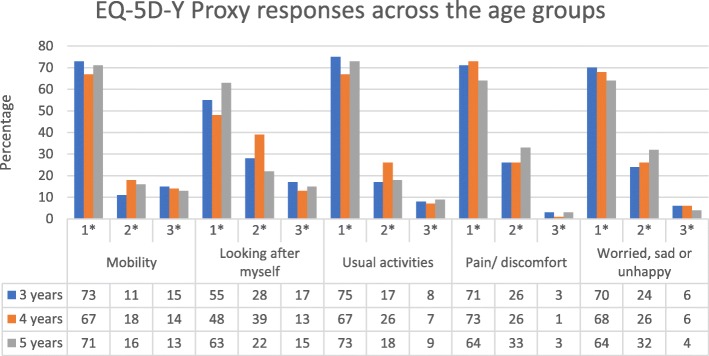
The distribution of responses to EQ-5D-Y Proxy 1 dimensions at baseline by age group. 1* = no problems, 2* = some problems, 3* = a lot of problems. 3 years N = 105; 4 years N = 98; 5 years N = 118

The distribution of problems on each dimension, apart from *Looking after Myself*, was similar for each age group (Fig. 1), and the percentage reporting no problems ranged from 64% in the *Worried Sad or Unhappy* 5-year olds to 75% of the youngest group in *Usual Activities*. *Looking after Myself* had the greatest variability between age groups and had the lowest rate of no problems reported (48–63%). No progressive age differential was discerned, and the 4-year olds had a slightly higher rate of problems than the other groups (although this was not a significant difference).

Neither the mean EQ-5D scores nor the PedsQL Total scores as depicted in Table 2 were significantly different between the age groups.

**Table 2:** Mean (SD) scores for EQ-5D-Y and PedsQL scores by age group

	3 years (***n*** = 105)		4 years (***n*** = 98)		5 years (***n*** = 118)			
	Mean	SD	Mean	SD	Mean	SD	ANOVA	p-value
EQ-5D-Y Latent Score	−1.18	1.53	−1.19	1.45	−1.35	1.54	F(2.316) = 0.115	0.899
EQ-5D-Y VAS	77.24	19.55	81.55	19.53	78.07	20.39	F(2.318) = 1.342	0.263
EQ-5D Dolan	0.66	0.36	0.67	0.34	0.67	0.36	F(2.316) = 0.001	0.999
PedsQL Total	63.49	11.28	63.81	12.95	63.19	11.20	F(2,318) = 0.879	0.416

EQ-5D-Y VAS score and PedsQL Total are measured between 0 and 100 with a higher score indicating a better HRQoL

### Test Retest Reliability

There were 101 CI children who participated in the study, of these a second measure of the EQ-5D-Y Proxy was captured for 85. Sixteen of the participants were lost to follow up as they did not answer the follow up telephone call.

Test retest reliability for the 3-year olds was insignificant for two individual dimensions of *Pain or Discomfort* and *Worried Sad or Unhappy* but the overall summary scores were more acceptable (Table 2). For the 4-year olds the *Usual Activities* dimension had insignificant, very low agreement and the dimension of *Worried Sad or Unhappy* had insignificant fair agreement, but the sample is limited with only 18 respondents. For the 5-year olds the dimension *Worried Sad or Unhappy* had insignificant poor agreement. Good agreement is observed for EQ-5D Dolan and EQ-5D-Y VAS, but not for EQ-5D-Y Latent score, with ICC and r < 0.7 in the three age groups: 0.512 and 0.517; 0.244 and 0.235; 0.587 and 0.591. The EQ-5D Latent score and Dolan score for the middle age group (4-year olds) showed lower reliability, but this group only included 18 participants. There is no clear evidence that test retest reliability differs by age in a systematic way, but the dimension scores do indicate issues that should be examined more closely (Table 3).

**Table 3:** Test retest reliability for EQ-5D-Y dimension scores and summary scores by age group in a group of chronically-ill children

		3 years (***n*** = 31)	4 years (***n*** = 18)	5 years (***n*** = 36)
**Dimension Scores**				
Mobility	**κ**	0.883 **	0.800 **	0.762 **
Looking after Myself	**κ**	0.725 **	0.406*	0.601 **
Usual Activities	**κ**	0.264 *	0.100	0.526 **
Pain/ discomfort	**κ**	0.265	0.357	0.446 *
Worried, Sad or Unhappy	**κ**	0.028	1.00 **	0.166
**Summary Scores**				
EQ-5D-Y Latent score	**ICC**	0.512**	0.244	0.587 **
	**r**	0.517 **	0.235	0.591**
EQ-5D Dolan	**ICC**	0.797 **	0.544 **	0.733 **
	**r**	0.799 **	0.545 *	0.736 **
EQ-5D-Y VAS	**ICC**	0.807 **	0.926 **	0.722 **
	**r**	0.699 **	0.857 **	0.827 **

*κ* Kappa, *ICC* Intraclass Correlation Coefficient*, r* Pearson’s correlation. * = *P*< 0.05, ** = *P*< 0.001

### Concurrent Validity of the EQ-5D-Y Proxy and PedsQL

Table 4 indicates that the EQ-5D-Y *Mobility* score and PedsQL Physical Score were logically ordered for all age groups with a large significant effect size. The EQ-5D-Y dimension of *Usual Activities* and the PedsQL Social scores had medium significant effect sizes for the 3 year and 4 year groups. However, in the 5 year group the effect size was small and insignificant with some problems on the EQ-5D-Y *Usual Activity* domain had a lower PedsQL Social mean score (worse HRQoL) than a lot of problems on the EQ-5D-Y. Although the effect size was large and medium in the 4 year and 5 year group respectively for the EQ-5D-Y *Worried, Sad or Unhappy dimension* the PedsQL Emotional scores were not ordered in the 3 year or 5 year group. In the 3 year group no problems on the EQ-5D-Y *Worried, Sad or Unhappy* had a lower PedsQL Emotional score (worse HRQoL) than some problems on the EQ-5D-Y. Similarly in the 5 year old group a lot of problems on the EQ-5D-Y *Worried, Sad or Unhappy* dimension had a higher PedsQL Emotional score (better HRQoL) than either no problems or some problems on the EQ-5D-Y.

**Table 4:** Summary table of mean PedsQL dimension scores by age and EQ-5D-Y dimension

	3 years	4 years	5 years
	(***n*** = 105)	(***n*** = 98)	(***n*** = 118)
EQ-5D-Y Dimensions	Mean (95% CI)	Mean (95% CI)	Mean (95% CI)
PedsQL Physical Score			
Mobility			
1	68.37 (10.97 to 15.11)	70.30 (10.07 to 14.24)	68.57 (8.96–12.16)
2	48.33 (14.59 to 34.97)	55.27 (11.54 to 23.06)	65.26 (11.78–23.05)
3	34.68 (22.59 to 47.32)	25.71 (18.31–40.69)	25.00 (18.20–39.20)
Effect Size	**0.357 (*p*** **< 0.001)**	**0.526 (*p*** **< 0.001)**	**0.527 (*p*** **< 0.001)**
PedsQL Social Score			
Usual Activities			
1	70.04 (7.87 to 10.79)	69.57 (7.33 to 10.36)	67.44 (9.73 to 13.17)
2	67.13 (9.46 to 18.91)	63.00 (10.47 to 18.66)	63.49 (10.37 to 19.58)
3	59.37 (14.26 to 43.89)	60.71 (15.72 to 53.72)	67.42 (4.08 to 10.25)
Effect Size	**0.066 (*p*** **= 0.030)**	**0.080 (*p*** **= 0.018)**	0.018 (p = o.351)
PedsQL Emotional Score			
Worried Sad or Unhappy			
1	63.68 (11.57 to 16.03)	65.48 (9.49 to 13.40)	65.67 (9.91 to 13.71)
2	65.25 (8.58 to 15.28)	60.75 (11.31 to 20.16)	56.25 (14.98 to 23.78)
3	56.25 (3.49 to 13.71)	39.58 (18.07–71.02)	67.50 (3.13 to 15.03)
Effect Size	0.024 (*p* = 0.296)	**0.180 (*P*** **< 0.001)**	**0.095 (*p*** **= 0.003)**

PedsQL Physical, social and emotional items are scored from 0 to 100, A higher PedsQL score indicates a better HRQoL. Effect Size is calculated with Partial Eta-Squared and significant results, with a medium or large effect are bolded

Only comparable dimensions of the EQ-5D-Y proxy and PedsQL were included. As no item on EQ-5D-Y assess school functioning this was not included. Similarly, no items on the PedsQL assess *Looking after Myself* or *Pain or Discomfort*.

The EQ-5D-Y Latent score, Dolan tariff and VAS had fair to moderate but significant correlations with PedsQL total score, with the exception of the VAS in 4-year olds. There was no systematic evidence that these relationships were weaker for the younger age group compared with the older children (Table 5).

**Table 5:** Summary table of EQ-5D-Y concurrent validity

		3 years (***n*** = 105)	4 years (***n*** = 98)	5 years (***n*** = 118)
EQ-5D-Y Latent score	PedsQL Total	0.373**	0.458**	0.244*
EQ-5D Dolan	PedsQL Total	0.454**	0.529**	0.353**
EQ-5D-Y VAS	PedsQL Total	0.322**	0.092	0.472**

Pearson’s r: * = P < 0.05, ** = P < 0.001

### Known Group Validity

**Fig. 2:**
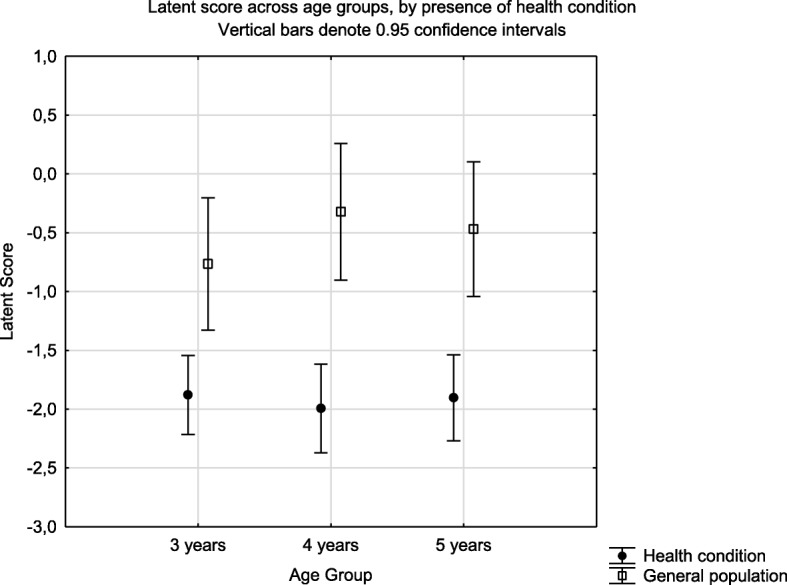
Known group validity of the EQ-5D-Y Latent score across age groups and health condition. Health condition implies attendance at a health institution for acute or chronic illness

**Fig. 3:**
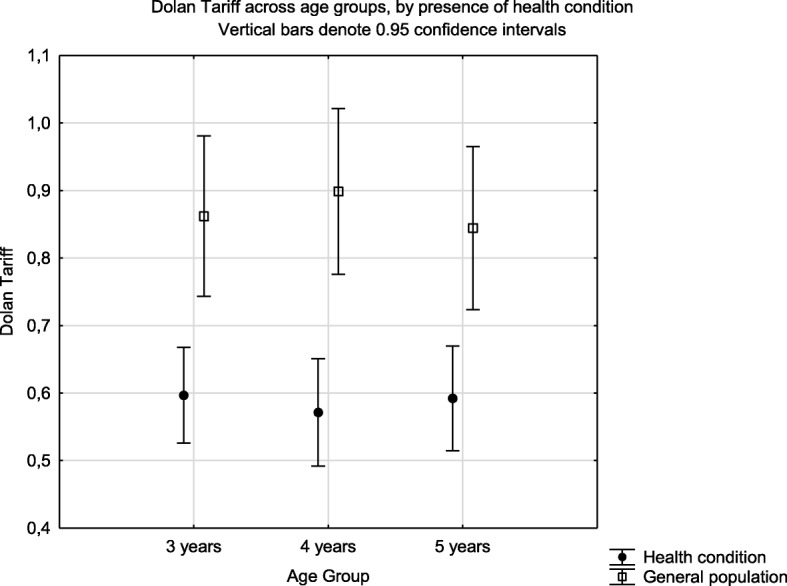
Known group validity of the EQ-5D Dolan tariff across age groups and health condition. Health condition implies attendance at a health institution for acute or chronic illness

**Fig. 4:**
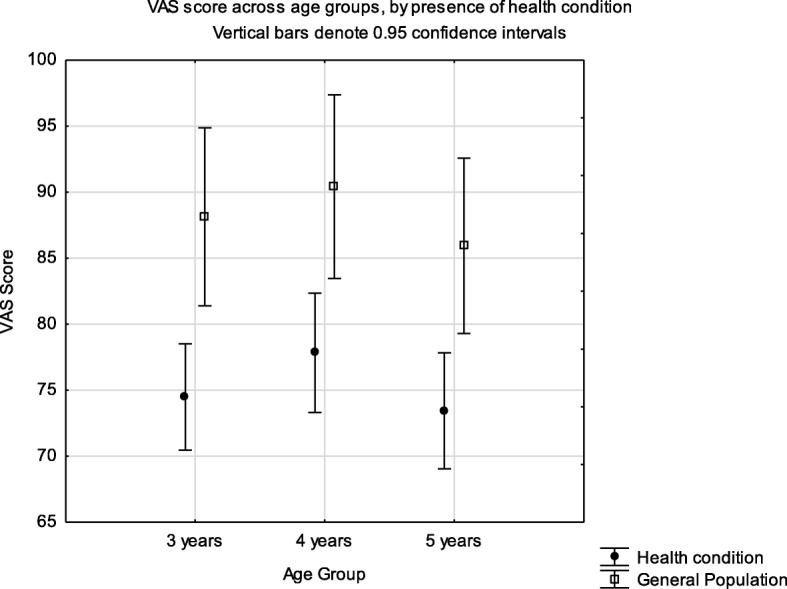
Known group validity of the EQ-5D-Y VAS score across age groups and health condition. Health condition implies attendance at a health institution for acute or chronic illness

Known group validity of the EQ-5D-Y latent score when analysed by the presence of health condition identified a significant difference in the mean scores (F = 50.36, *p* < 0.001) (Fig. 2). The same effect was seen for the single index scores estimated using the Dolan tariff (F = 45.16, *p* < 0.001) (Fig. 3) and EQ-5D VAS (Fig. 4) (F = 30.0, *p* < 0.001). There was no interaction effect between presence of a health condition and the age group for latent value (F = 0.673, *p* = 0.511); Dolan Tariff (F = 0.296, *p* = 0.744) or VAS score (F = 0.025, *p* = 0.975).

### Suitability of Dimensions as Recorded by the Caregivers

It was hypothesised that the caregivers would report a higher number of dimensions as not being suitable for their child in the younger age groups.

Caregivers’ views on the suitability of dimensions were explored to better understand the content validity and appropriateness of the EQ-5D-Y. The dimension of *Looking after Myself* was reported across the age-groups as unsuitable but was highest in the 3-year olds with 27% of caregivers reporting it unsuitable (Table 6). All the caregivers reported that the difficulty in answering the question was due to the age appropriate demands of washing and dressing and they felt that their child should not yet be able to complete the tasks independently.

**Table 6:** Percentage of Respondents reporting that dimensions were not suitable for their child

	3 Years (***n*** = 105) N (%)	4 Years (***n*** = 98) N (%)	5 Years (***n*** = 118) N (%)
Mobility	0 (0)	1 (1)	1 (1)
Looking after Myself	28 (27)	14 (14)	15 (13)
Usual Activities	4 (4)	3 (3)	1 (1)
Pain/ discomfort	1 (1)	1 (1)	0 (0)
Worried, Sad or Unhappy	5 (5)	0 (0)	1 (1)
VAS	2 (2)	5 (5)	4 (3)

## Discussion

The age groups were recruited using the same methods with a mix of children from the general population, children with chronic diseases and children with acute disease. Some differences between the three age groups emerged, particularly the data from the 4-year olds with a higher number of problems reported in the *Mobility*, *Looking after Myself* and *Usual Activities* dimensions. This group had a higher proportion of AI children which may account for this difference as previous research on older children that found AI children self-report high rates of problems in *Mobility* and *Usual Activities* [26, 27]. These differences were however only reflected at a dimension level as the mean scores for both the EQ-5D-Y Proxy and the PedsQL did not show differences between the age groups.

The test retest reliability of the EQ-5D-Y Proxy was similar to previous studies investigating the reliability of the EQ-5D-Y [16, 27, 28] with regards to dimension scores, and summary scores for all age groups. The 3-year olds had poor reliability with two of the dimensions (*Pain or Discomfort* and *Worried Sad or Unhappy*) which are less observable and not preferred for proxy completion [9]. The 4-year olds had poor reliability on two dimensions (*Usual Activities* and *Pain or Discomfort*) which should be explored further as it’s possible that this reflected the small number of 4-year olds who participated in the retest reliability. The test retest reliability of the summary scores provides an indication of how the measure may work when used in an evaluation. Unfortunately, this analysis is limited because there is no scoring system for the EQ-5D-Y that allows for the estimation of a single index score for the estimation of quality adjusted life years (QALY). For this reason, we present latent scoring system which is based on Discrete Choice Experiment (DCE) valuation technique by Mott et al. (2019) [12]. We also present analyses where data were scored using the Dolan algorithm which was developed for the adult version, EQ-5D-3 L [23]. This is limited because the EQ-5D-3 L has slightly different questions to the EQ-5D-Y although conceptually they are similar issues. The Dolan scores were further valued using time trade-off for the adult population and not considering children. The Dolan scores are presented merely to give an indication of what a time trade off based scoring system might produce when it becomes available. Thus, the test retest reliability results provide an important indication of the measurement properties of the EQ-5D-Y in these young children as at a summary score level the EQ-5D-Y appears to work as well in 3-year olds as it does in 5-year olds. The test-retest results are however limited as 15% of the follow-up calls were unanswered. It is recommended that future studies include a larger sample of participants for test-retest analysis to allow for this discrepancy.

Previous research comparing the EQ-5D-Y VAS and the PedsQL Total score in Italy showed similar results for concurrent validity with a weak to moderate correlation in a sample of children aged 8–15 years from the general population and children suffering from Acute Lymphoblastic Leukaemia [28]. A younger 4-year old sample showed concurrent validity to the PedsQL for the EQ-5D dimension scores, but not the VAS score. The comparable dimensions on the PedsQL and EQ-5D-Y only showed concurrent validity across all age groups for the physical dimension. The social score for PedsQL and the EQ-5D-Y *Usual Activity* Score did not show logical increment of the scores’ mean between the two instruments in the 5 year old group. This could indicate that the activities described are not all suitable for this age group. The PedsQL emotional score for the PedsQL and EQ-5D-Y *Worried, Sad or Unhappy* similarly showed an illogical increment of scores’ means between the two instruments in both the 3 and 5 year old groups*.* This could be attributed to the EQ-5D-Y dimension not giving any reference to observable behaviour of being worried, sad or unhappy in the relevant age group, but relies on inference from the proxy [9].

In the present study the EQ-5D-Y showed good known-group validity with significant differences for all age groups in mean summary scores between children with and without a known health condition. This did not vary by age group.

The *Looking after Myself* dimension was singled out as being the most difficult to respond to appropriately most especially in the 3 year old group. This was further reflected in the incongruence between this dimension and the other four, with the greatest frequency of caregivers reporting a problem with *Looking after Myself* across the age groups. This in stark contrast to other studies with older children, where it is usually the dimension with the least reported problems [16, 27, 28]. This impacts the content validity of the EQ-5D-Y for use in younger children, most notably in the youngest age group of 3-year olds. Consideration needs to be given to the adaptation or deletion of this dimension for use in the 3- year old group. The general population group was from the same geographical catchment area as the tertiary paediatric hospital. The issues found seemed to be reflective of the age groups included however, the results cannot be generalised to the greater Western Cape region as no data on race, home language or socio-economic status were collected for comparison to the general population of the Western Cape.

## Conclusion

Based on the above results and discussion, we suggest that the dimension of *Looking after Myself* is problematic for these young children but most notably so in the 3 year old group. Further work is needed in exploring either the adaptation of the *Looking after Myself* dimension or discarding it in the younger age groups. If one considers the summary scores of the EQ-5D-Y Proxy version 1 it appears to work well. Known group validity was demonstrated. Concurrent Validity was established on a composite level but not on an individual dimension level, further suggesting that revision of *Usual Activities and Worried, Sad or Unhappy* is warranted The observable dimensions (*Mobility*, *Looking after Myself* and *Usual Activities*) demonstrated stability over time, but the inferred dimensions (*Pain or Discomfort* and *Worried Sad or Unhappy*) were less stable, which is to be expected and consistent with proxy HRQoL research generally and consideration may need to be given to framing it from an observable perspective.

## Data Availability

The datasets used and/or analysed during the current study are available from the corresponding author on reasonable request.

## Abbreviations

AI: Acutely-ill
CI: Chronically-ill
GP: General Population
HRQoL: Health Related Quality of Life
ICC: Intra-class correlation coefficient
PedsQL: Paediatric Quality of Life Measure
QALYs: Quality Adjusted Life Years
VAS: Visual Analogue Scale
